# Evaluation of early chemotherapy response by combining static- and dynamic [^18^F]FDG-PET with diffusion-weighted MRI in subcutaneous patient-derived endometrial cancer mouse models

**DOI:** 10.1186/s13550-025-01235-5

**Published:** 2025-04-18

**Authors:** Heidi Espedal, Jenny M. Lyngstad, Hege F. Berg, Marta E. Hjelmeland, Kristine E. Fasmer, Camilla Krakstad, Ingfrid S. Haldorsen

**Affiliations:** 1 Department of Clinical Medicine, University of Bergen, Bergen, Norway; 2 Mohn Medical Imaging and Visualization Centre, Department of Radiology, Haukeland University Hospital, Bergen, Norway; 3 Western Australia National Imaging Facility, The University of Western Australia, Perth, Australia; 4 Centre for Cancer Biomarkers, Department of Clinical Science, University of Bergen, Bergen, Norway; 5 Department of Obstetrics and Gynecology, Haukeland University Hospital, Bergen, Norway

**Keywords:** Preclinical imaging, Treatment response, Dynamic PET, Endometrial cancer, Organoid models, Chemotherapy, Diffusion-weighted MRI, Metabolic rate of glucose

## Abstract

**Background:**

The combination of carboplatin and paclitaxel is the standard chemotherapy for treatment of high-risk and recurrent endometrial cancer. Evaluation of treatment response by diagnostic imaging is routinely carried out months after start of treatment, and is based on changes in tumor size or appearance of new metastases. The aim of this study was to evaluate early chemotherapeutic response in two subcutaneous endometrial cancer mouse models generated from patient-derived organoids using static- and dynamic [^18^F]fluorodeoxyglucose ([^18^F]FDG) positron emission tomography (PET) and diffusion-weighted (DW) magnetic resonance imaging (MRI). Mice were injected bilaterally with endometrioid endometrial cancer grade 3 (EEC G3), International Federation of Gynaecology and Obstetrics (FIGO) stage 3C1 (Model A) or stage 1B (Model B) organoids (*n* = 15 mice). The mice were randomized into treatment (combined carboplatin and paclitaxel, n_A_=8 / n_B_=6 tumors) or control (saline, n_A_=8 / n_B_=8 tumors) groups. During tumor progression, the mice underwent T2-weighted (T2w) MRI, DW-MRI and dynamic [^18^F]FDG-PET at baseline/Day 0 (start of treatment), Day 3 (early) and Day 10 (endpoint) using a sequential PET-MRI small-animal scanner.

**Results:**

At endpoint, tumor volumes at T2w-MRI (vMRI) were lower in the treatment groups in both models (*p* ≤ 0.029). The tumor metabolic rate (MR_FDG_) from dynamic PET, was significantly lower in the treatment group at the early timepoint (Day 3) and at the endpoint in Model A (*p* ≤ 0.042). In Model B, MR_FDG_ was similar for both groups at Day 3 and at endpoint (*p*≥0.217). The 10 tumor voxels with the highest standardised uptake value (SUV_10_) from static [^18^F]-FDG-PET was significantly lower at endpoint in the treatment groups in both models (*p* ≤ 0.041), but not at the early timepoint (*p*≥0.083). Similarly, the tumor apparent diffusion coefficient (ADC_mean_) was significantly higher indicating treatment response at endpoint for treatment groups in both models (*p* ≤ 0.036).

**Conclusions:**

Multimodal imaging is feasible for evaluation of early signs of treatment response in preclinical subcutaneous endometrial cancer models. The novel MR_FDG_ dynamic PET imaging parameter seems most promising for detecting very early treatment response following chemotherapy.

**Supplementary Information:**

The online version contains supplementary material available at 10.1186/s13550-025-01235-5.

## Background

Endometrial cancer is the most common gynecological cancer in high-income countries and the incidence is increasing [1, 2]. Surgery is the primary treatment and is curative in most women with early stage and low-risk disease. However, about 15% of these patients experience recurrence after surgery and typically have poor prognosis [2, 3]. Adjuvant chemotherapy with paclitaxel and carboplatin is recommended for patients with high-risk histology, advanced stage disease and recurrence [4]. Unfortunately, chemotherapy treatment is associated with toxic side-effects and only moderately improves survival in advanced endometrial cancer [5–7]. Evaluation of chemotherapy response is routinely carried out months after start of treatment using conventional imaging methods such as abdominal computed tomography (CT) or pelvic magnetic resonance imaging (MRI). The Response Evaluation Criteria in Solid Tumors (RECIST) standards are used to evaluate response, and reports on changes in tumor size or appearance of new metastases [8]. Earlier detection of treatment response may allow early treatment adjustments and reduction of unnecessary side-effects in poor- or non-responding women, improving overall clinical outcomes [5].

Small-animal, non-invasive PET-MR imaging may be used for preclinical treatment studies, offering simultaneous and longitudinal assessments of metabolic-, microstructural- and anatomical tumor changes, providing comprehensive insights into treatment response. [^18^F]FDG-PET is widely used in primary diagnostics and follow-up of many cancer types. Malignant tumor cells typically have elevated glucose metabolism compared to normal cells, leading to increased [^18^F]FDG uptake that is detectable and quantifiable in the PET images using standardised uptake values (SUVs). Furthermore, decreased [^18^F]FDG tumor uptake is associated with treatment response in several cancers. While [^18^F]FDG-PET detects increased glucose metabolism in malignant tumors, DW-MRI is used to quantify and depict diffusion of water molecules reflecting microstructural tissue characterisation. Malignant tumors usually have higher density which induces restricted diffusion. The DW images are acquired by applying different diffusion weighting (b-values), and apparent diffusion coefficient (ADC) maps are derived for quantification of diffusion properties. Treatment-induced cell death is known to reduce tumor cellularity, this effect can be depicted as normalisation of diffusion characteristics with increased tumor ADC values [9]. Preclinical studies in breast and ovarian cancers have shown that [^18^F]FDG-PET and/or DW-MRI may detect early treatment responses to chemotherapy, detecting metabolic and cellular changes prior to reduction in tumor volume [10–13]. Additionally, a preclinical breast cancer study has reported that dynamic [^18^F]FDG-PET parameters were better at assessing chemotherapy response compared to static SUVs [14]. With the recent introduction of long-axial field of view clinical PET scanners enabling whole-body dynamic imaging, there is increasing interest in the potential benefits of using dynamic imaging in cancer patient management [15–17].

We have previously shown the feasibility of MRI and dynamic [^18^F]FDG-PET in orthotopic endometrial cancer models [18], and the use of MR radiomics to detect early treatment response in these models [19]. To our knowledge, PET-MR imaging has not been performed to assess treatment response in preclinical endometrial cancer models. In the present study, we aim to evaluate early signs of imaging treatment response using combined DW-MRI and static- and dynamic [^18^F]FDG-PET in clinically relevant, organoid-based subcutaneous endometrial cancer models following treatment with carboplatin and paclitaxel.

## Methods

The use of human tumor material was approved by the Regional Ethical Committee of Western Norway (*REK Vest*, Approval ID 2015/2333 and 2018/548). Animal experiments were approved by The Norwegian Food Safety Authority (*Mattilsynet*, approval ID 20194) in accordance with guidelines from the Federation of European Laboratory Animal Science Association (FELASA). The animal facility was accredited by the Association for Assessment and Accreditation of Laboratory Animal Care (AAALAC).

### Animal model

Two organoid models were used in the study. Biopsies from patients diagnosed with endometrioid endometrial cancer grade 3 (EEC G3) and International Federation of Gynaecology and Obstetrics (FIGO) stage 3C1 (Model A) or 1B (Model B) were used to generate organoids as described earlier [20]. Model A and B both belong to the DNA mismatch repair deficiency (MMR-D) molecular subtype (ProMiSE classifier), but differ in expression of estrogen and progesterone receptors [20]. NOD/SCID IL2rγ^null^ (NSG) female mice at 8–10 weeks age, were subcutaneously injected with organoids (0.5 × 10^6^ cells immersed 1:1 in matrigel) in the left and right dorsal flank under sevoflurane (2% in oxygen) anesthesia. Model A was injected in 8 mice (16 tumors total), and Model B was injected in 7 mice (14 tumors total). Before including the mice in the sequential imaging scheme, tumor growth was monitored biweekly by palpation. The mice were monitored throughout the study for presence of disease symptoms including lethargy, ataxia and weight loss following a scoring system, and were sacrificed when the score met the predetermined threshold or at the study endpoint. The number of tumors included for image analyses per model, group and timepoint is listed in Supplementary Table 1, Additional File 1.

### Study design

**Fig. 1:**
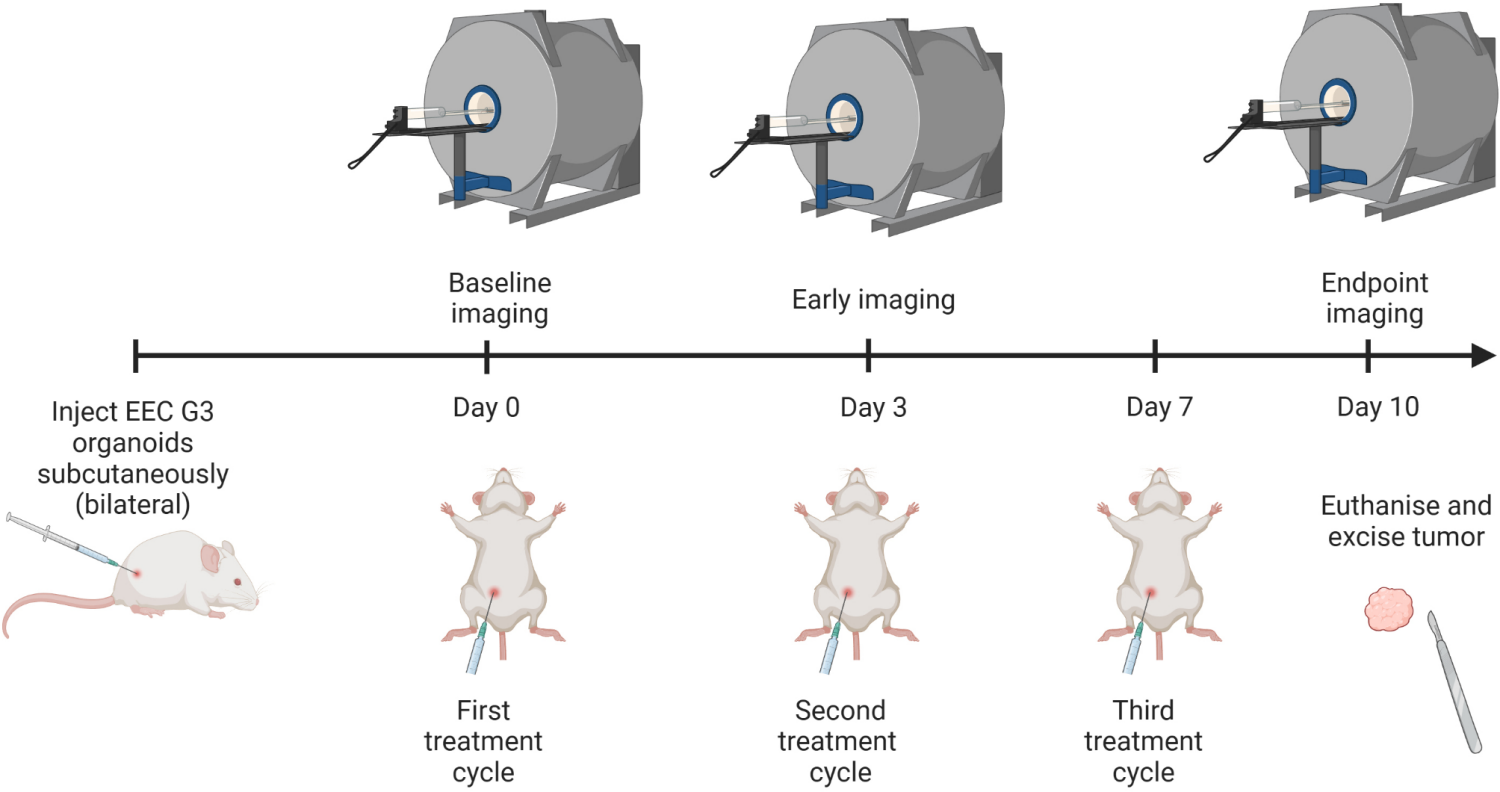
Study design. Study overview and timeline for both EEC G3 O-PDX models (Model A and B). All mice underwent three MRI-PET imaging sessions, and three treatment cycles of either Carbo-PTX (treatment group) or saline (control group) starting when the biggest tumor in each mouse was approximately 100 mm^3^. The first treatment was given immediately following the baseline imaging (Day 0). Made with Biorender.com. **Abbreviations**: Carbo-PTX Carboplatin-paclitaxel, EEC G3 endometrioid endometrial cancer grade 3, MRI magnetic resonance imaging, O-PDX Organoid-based patient-derived xenograft model, PET: positron emission tomography

The study design is illustrated in Fig. 1. All mice were imaged at Day 0 (baseline), Day 3 (early timepoint) and at Day 10 (endpoint). The mice were randomized into treatment or control groups when the largest tumor in each mouse was ~ 100 mm^3^ (median size = 78.7 [interquartile range (IQR) 58.8-100.3] mm^3^). The baseline tumor size was based on pilot experiments balancing growth rate, treatment response and animal welfare. The treatment groups received combined carboplatin (15 mg/kg) / paclitaxel (12 mg/kg) (Carbo-PTX) and the control groups received saline (100 µl) intraperitoneally at baseline (immediately following the imaging at Day 0), at Day 3 (post-imaging) and at Day 7, for a total of 3 treatment cycles. The chemotherapy dosage and frequency has been optimised in previous studies, is well tolerated [18–20] and translatable to clinical doses [21]. The mice were euthanized by cervical dislocation immediately after the endpoint imaging, and tumors were dissected for tissue analysis.

### MRI-PET acquisition and image reconstruction

Images were acquired on a small-animal, 7 Tesla sequential MRI-PET scanner (MRS DRYMAG 7017 with PET Clip-On 803 ring, MR Solutions, United Kingdom). Prior to each imaging session, mice were fasted overnight to minimise physiologic [^18^F]FDG uptake. Mice were anesthetized by sevoflurane (2%) mixed in oxygen for imaging and placed on a heating pad (37^◦^ C). A custom-made cannula consisting of a fine insulin-needle (0.7 mm) connected to a polyethylene tubing (PE10) for dynamic [^18^F]FDG intravenous injection was inserted in the tail vein prior to animal placement in the scanner. Mice were placed in pairs on a dedicated dual-mouse bed using a 65 mm diameter volume coil. T2-weighted images were acquired coronally (TE/TR 45/3500 ms, 3 averages, matrix 239 × 256, field of view 30 × 60 mm, slice thickness 0.9 mm, resolution 0.25 × 0.24 mm). Coronal DW-images (TE/TR 27/5000 ms, 1 average, 4 directions, matrix 100 × 114, field of view 58 × 36 mm, slice thickness 1 mm, slice gap 0.5 mm, resolution 0.32 × 0.58 mm) were generated using b-values of 0 and 1000 mm^2^/s. Immediately following the MRI sequences, the mice were automatically positioned in the PET ring and [^18^F]FDG was manually injected as a bolus (Mean injected activity = 7.7 ± 1.0 MBq) following the start of the 1-hour list-mode acquisition. Respiratory rate was monitored throughout the imaging and temperature was kept constant at 37 °C by heated water flowing through the animal bed.

Static PET images were reconstructed using the list-mode data from 30 to 60 min post [^18^F]FDG injection. Dynamic PET images (60 min) were reconstructed into the following time frames: 5 × 2 s, 5 × 10 s, 2 × 120 s, 3 × 300 s, 4 × 600 s [18]. All reconstructions were performed applying a maximum likelihood estimation method (MLEM) algorithm by 2 iterations and 32 subsets as recommended by the manufacturer, resulting in 0.42 × 0.42 × 0.42 mm voxel size corrected for randoms and scatter. Images were not corrected for attenuation.

### MR image analyses

The whole tumor was manually segmented on the coronal T2-weighted images using ITK-SNAP software (Version 3.8). The anatomic tumor volume (vMRI) was automatically calculated by summing the segmented volumes from all slices depicting tumor tissue. Apparent diffusion coefficient (ADC) parametric maps were generated from the DWI using VivoQuant software (Version 2020, Invicro, MA, USA) and the standard equation S(b) = S_0_ e^− b(ADC)^ where S represents the signal and b represents the b-value. The ADC maps were analysed in PMOD software (Version 4.1, PMOD Technologies Ltd., Zürich, Switzerland) and the average tumor ADC (ADC_mean_) was measured by placing 2–3 circular regions of interest with 1–2 mm radius (dependent on tumor size) in representative areas of the tumor. All image analyses were carried out blinded to treatment groups.

### PET image analyses

We applied a spherical volume of interest (VOI) including the whole tumor, and automatically selected the 10 hottest voxels within the VOI to calculate the average static [^18^F]FDG uptake (SUV_10_). We used the mean liver uptake (1 mm radius spherical VOI placed in the right liver lobe) to normalise the tumor signal (Tumor SUV_10_ /Liver SUV_mean_) and minimise variability [22, 23]. SUV_max_ (the single hottest voxel) was obtained for comparison with SUV_10_.

Dynamic PET analyses were performed as previously described [18]. The SUV_10_ tumor voxels from the static images, and an image-derived input function (IDIF) from the vena cava from the dynamic images were used as input, which is a minimally invasive method to obtain the input function and shown to be robust in rodents [24]. We extracted the tumor influx constant (K_i_) using the Patlak model and calculated the tumor metabolic rate of glucose (MR_FDG_) using the equation MR_FDG_=K_i_ x (blood glucose/lumped constant). We used 0.6 as the lumped constant [25], and the mean blood glucose measured in selected mice (mean = 4.9 mmol/l, ± 1.6, *n* = 23 series) for all analyses, as this was not measured in all mice. The tumor MR_FDG_ was normalised to the liver uptake (MR_FDG_ / Liver SUV_mean_). All Patlak slope fits resulted in < 20% standard error (mean = 7.7 ± 3.8). Both the static and dynamic PET analyses were carried out in PMOD.

### Histological analyses

Hematoxylin and eosin (H&E) tissue slides from the dissected tumors were prepared and processed as previously described [18]. Semi-automatic counting of nuclei was performed using the open-access software QuPath (Version 0.5.1) [26] in 1–3 circular regions of interest (dependent on tumor size) covering the tumor. Cell counting was done blinded to treatment groups.

### Statistical analyses

Data normality was assessed for all datasets using the Shapiro-Wilk test. An unpaired t-test or Mann-Whitney test was used to compare treatment groups at the different time-points. A paired t-test or a Wilcoxon matched pairs signed rank test was used to assess paired response between the baseline and the early timepoint, or between the baseline and the endpoint within the groups. A Pearson- or Spearman *r* was calculated to assess correlation between the PET parameters. Statistical analyses were conducted using GraphPad Prism (Version 10, GraphPad Software, CA, USA). *P*-values < 0.05 were considered statistically significant.

## Results

### Longitudinal tumor imaging characteristics

**Fig. 2:**
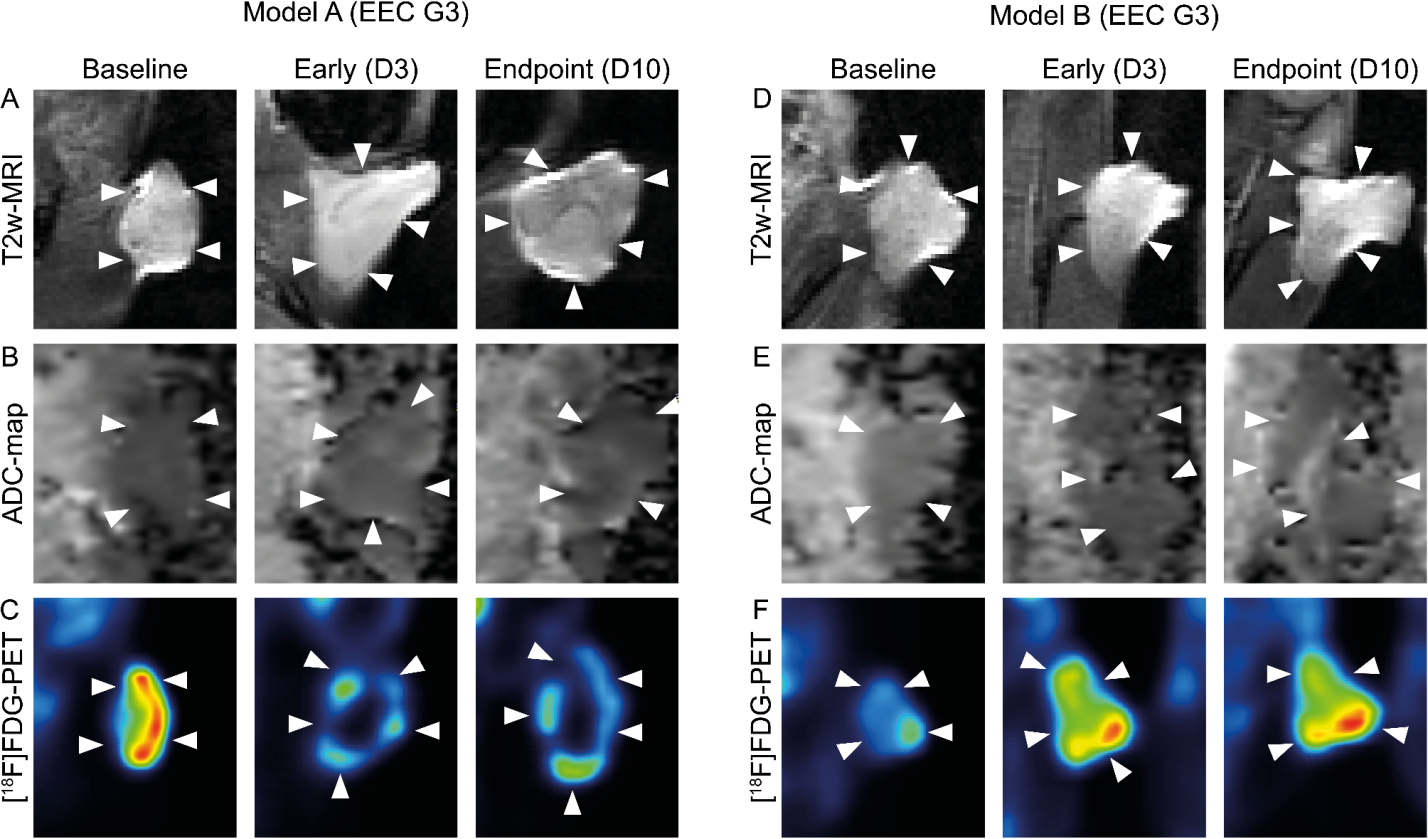
Representative tumor images. MRI and static [^18^F]FDG-PET images from a representative tumor from each model (control group) and timepoint. White arrows indicate tumor location. The vMRI for, EEC G3 Model A (A) was 78.1 mm^3^ (Baseline), 106.4 mm^3^ (Early) and 193.2 mm^3^ (Endpoint), For EEC G3 Model B (D) the vMRI was 96.8 mm^3^ (Baseline), 102.2 mm^3^ (Early) and 127.5 mm^3^ (Endpoint) mm^3^. Scale ADC-map: 0-0.0025 mm^2^/s. Scale PET: 0-3.5 SUV. **Abbreviations**: ADC apparent diffusion coefficient, D day, EEC G3 endometrioid endometrial cancer grade 3, [^18^F]FDG fluorodeoxyglucose, MRI magnetic resonance imaging, PET positron emission tomography, SUV standardised uptake value, T2w T2-weighted, vMRI MRI-derived tumor volume

Representative T2w-MRI, ADC-maps and static [^18^F]FDG-PET tumor images (from the control groups) for both EEC G3 O-PDX models at the three timepoints are shown in Fig. 2. The tumors displayed characteristic imaging features, depicted as hyperintense on the T2w-images and with distinct boundaries from adjacent normal tissue (Fig. 2A, D). All tumors were hypointense on the ADC-maps relative to the surrounding tissue (Fig. 2B, E) and had increased [^18^F]FDG tumor uptake on the static PET images (Fig. 2C, F). Tumors in Model A frequently appeared with a circular-shaped increased uptake pattern in the periphery of the tumor in the PET images (Fig. 2C), whilst this pattern was less common in Model B (Fig. 2F).

### Chemotherapy treatment results in decreased endpoint tumor vMRI in both models

**Fig. 3:**
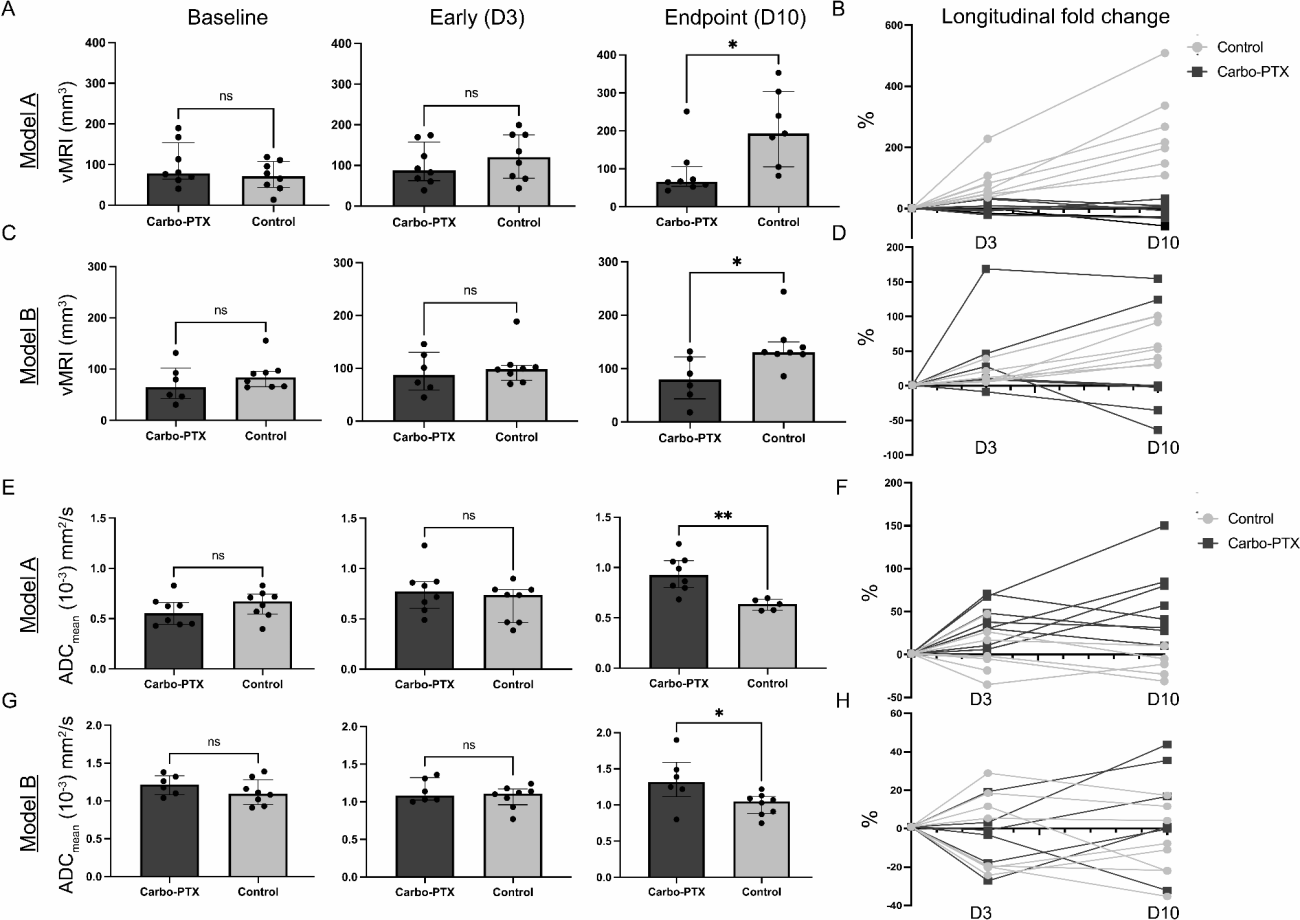
MRI-derived imaging markers. Quantification of vMRI and ADC_mean_ from the MR images from EEC G3 Model A (**A**-**B**, **E**-**F**) and EEC G3 Model B (**C**-**D**, **G**-**H**) at the different timepoints. Median and interquartile range are displayed for the bar graphs. Statistical significance (*p*-value < 0.05) is indicated by *. The fold change (%) was calculated from the baseline values. **Abbreviations**: ADC apparent diffusion coefficient, Carbo-PTX Carboplatin-paclitaxel, D day, DWI diffusion-weighted imaging, EEC G3 endometrioid endometrial cancer grade 3, ns not significant, vMRI tumor volume derived from T2w-MRI

Median baseline tumor vMRIs were similar for the control group (71.6 mm^3^ [IQR: 44.0-107.1] and the treatment group (78.4 mm^3^ [63.8-153.9], *p* = 0.440) in Model A (Fig. 3A). At the early timepoint (Day 3, after one treatment cycle), the median vMRI tended to be lower for the treatment group (87.4 [62.5-157.9] mm^3^) compared to the control group (120.6 [68.7-175.5], mm^3^, *p* = 0.380). At the endpoint and after a total of three treatment cycles, the median tumor vMRI was significantly lower in the treatment group (66.2 [53.8-105.5] mm^3^) compared to the control group (193.2 [104.8-303.4] mm^3^, *p* = 0.014). The vMRI longitudinal fold change of individual tumors compared to baseline vMRI is illustrated in Fig. 3B. Paired analysis within the groups showed significantly increased vMRI from baseline for the control group at the early timepoint (*p* = 0.001) and the endpoint (*p* = 0.002). *P*-values for all paired analyses are shown in Table 1.

In Model B, the median [IQR] vMRI was similar between the control- and treatment group both at baseline (control: 83.9 [65.8–95.9] mm^3^; treatment; 64.9 [42.6-102.3] mm^3^, *p* = 0.345). and at the early timepoint (control: 98.3 [77.6-105.6] mm^3^; treatment: 87.2 [59.1-130.4] mm^3^, *p* = 0.662) (Fig. 3C). At endpoint (Day 10) the median tumor vMRI was significantly lower for the treatment group (79.7 [43.3–122.0] mm^3^) compared to the control group (130.5 [127.3-150.2] mm^3^, *p* = 0.029). The vMRI longitudinal fold change of individual tumors compared to baseline vMRI is illustrated in Fig. 3D. Paired analysis within the groups showed significantly increased vMRI for the control group from baseline to early timepoint (*p* = 0.008) and to endpoint (*p* = 0.008) (Table 1).

**Table 1:** Longitudinal, paired analyses for all imaging parameters

	Model A (EEC G3)		Model B (EEC G3)	
	Carbo-PTX	Control	Carbo-PTX	Control
vMRI				
Early	0.840	*0.001*	0.151	*0.008*
Endpoint	0.641	*0.002*	0.640	*0.008*
ADC_mean_				
Early	*0.003*	0.794	0.458	0.565
Endpoint	*< 0.001*	0.155	0.393	0.180
SUV_10_				
Early	0.967	0.383	0.564	0.070
Endpoint	0.055	0.438	0.550	0.438
MR_FDG_				
Early	0.680	0.104	0.410	0.844
Endpoint	0.345	0.135	*0.028*	0.999

*P*-values from pairwise analyses within the treatment groups between baseline values and the early timepoint (Early), and between baseline values and the endpoint (Endpoint). Statistically significant *p*-values (*p* < 0.05) in *italics*

**Abbreviations**: ADC apparent diffusion coefficient, EEC G3 endometrioid endometrial cancer grade 3. MR_FDG_ metabolic rate of [^18^F]FDG, derived from dynamic imaging, SUV_10_ standardised uptake value of 10 hottest tumor voxels, vMRI tumor volume derived from T2w-MRI

### Tumor diffusion at endpoint is increased after chemotherapy in both models

Median [IQR] tumor ADC_mean_ was similar between the control- and treatment group in Model A, both at baseline (control: 0.67 [0.55–0.74]*10^− 3^ mm^2^/s; treatment: 0.56 [ 0.44–0.66]*10^− 3^ mm^2^/s; *p* = 0.313) and at the early timepoint (Day 3) (control: 0.74 [0.46–0.79]*10^− 3^ mm^2^/s; treatment: 0.77 [IQR 0.61–0.87]*10^− 3^ mm^2^/s *p* = 0.262) (Fig. 3E). However, at endpoint (Day 10) median tumor ADC_mean_ was significantly higher in the treatment group (0.93 [0.80–1.07]*10^− 3^ mm^2^/s) than in the control group (0.64 [0.58–0.69]*10^− 3^ mm^2^/s, *p* = 0.004) reflecting normalisation of diffusion properties in the tumors in the treatment group. The ADC_mean_ longitudinal fold change of individual tumors compared to baseline ADC_mean_ is shown in Fig. 3F. Paired analyses within the groups showed significantly increased tumor ADC_mean_ for the treatment group at the early timepoint (*p* = 0.003) and at the endpoint (*p* < 0.001) compared to the ADC_mean_ at baseline in Model A (Table 1).

In Model B, median [IQR] tumor ADC_mean_ was similar between the two groups at baseline (control: 1.10 [0.95–1.28]*10^− 3^ mm^2^/s; treatment: 1.22 [1.09–1.34]*10^− 3^ mm^2^/s, *p* = 0.266) and at the early timepoint (control: 1.11 [0.96–1.17] *10^− 3^ mm^2^/s; treatment: 1.09 [1.02–1.32]*10^− 3^ mm^2^/s, *p* = 0.369) (Fig. 3G). At endpoint, however, the tumor ADC_mean_ was significantly increased in the treatment group (1.32 [1.12–1.59]*10^− 3^ mm^2^/s) compared to the control group (1.05 [0.88–1.12]*10^− 3^ mm^2^/s *p* = 0.036) reflecting normalisation of diffusion properties in tumors in the treatment group. The ADC_mean_ fold change of individual tumors compared to baseline is illustrated in Fig. 3H.

### Endpoint SUV_10_ derived from static [^18^F]FDG-PET is decreased after chemotherapy in both models

**Fig. 4:**
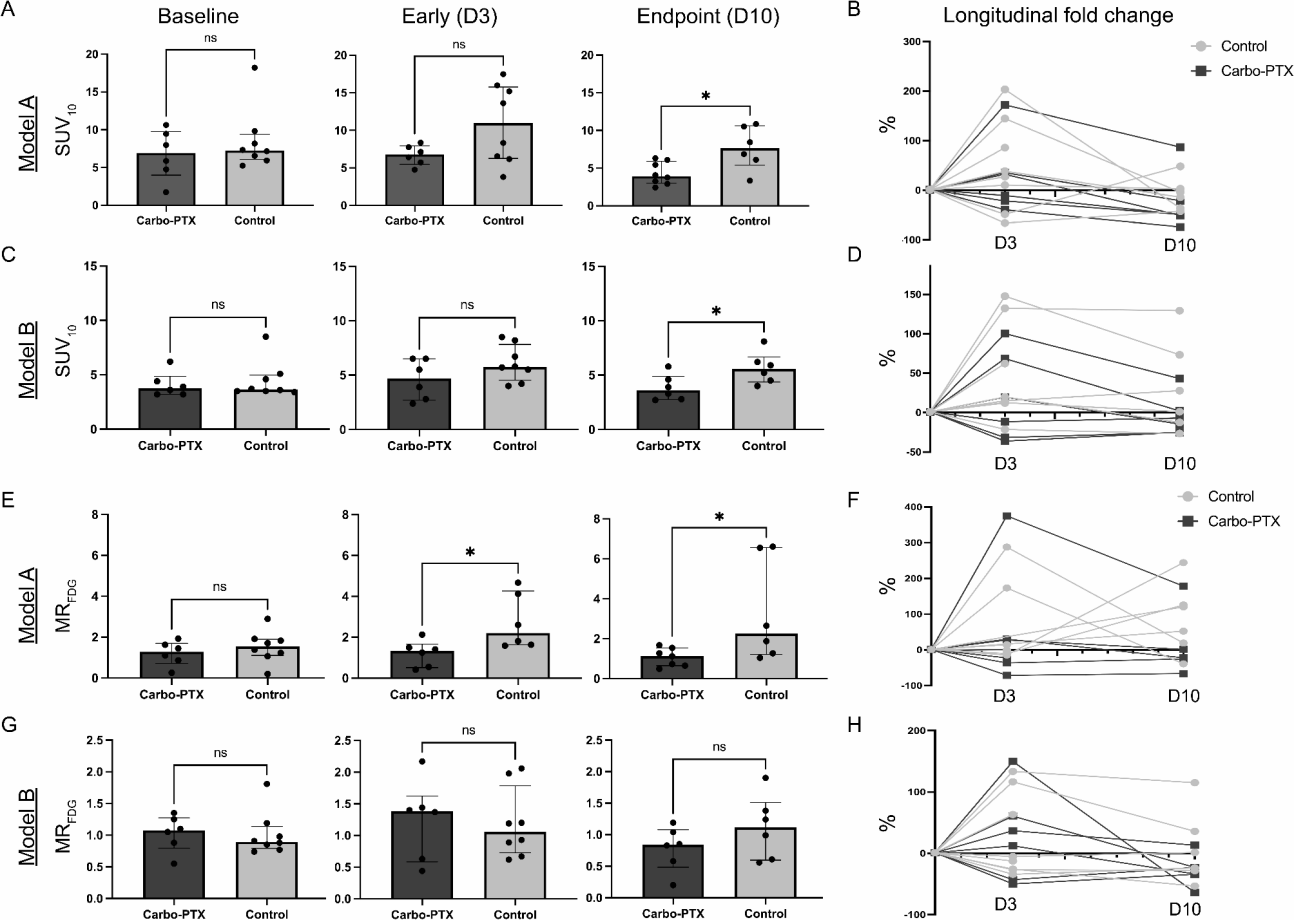
PET-derived imaging markers. Quantification of static and dynamic parameters derived from the PET images for EEC G3 Model A (**A**-**B**, **E**-**F**) and EEC G3 Model B (**C**-**D**, **G**-**H**) for the different timepoints. Median and interquartile range are displayed for the bar graphs. Statistical significance (*p*-value < 0.05) is indicated by *. The fold change (%) was calculated from the baseline values. **Abbreviations**: Carbo-PTX Carboplatin-paclitaxel, D day, EEC G3 endometrioid endometrial cancer grade 3, [^18^F]FDG fluorodeoxyglucose, MR_FDG_, metabolic rate of FDG, ns not significant, SUV_10_ standardised uptake value of 10 hottest tumor voxels

The median [IQR] tumor [^18^F]FDG uptake (SUV_10_) quantified from the static PET images was similar between the two groups in Model A at baseline (control: 7.2 [6.1–9.2]; treatment: 6.9 [4.0-9.7], *p* = 0.573), Fig. 4A. At the early timepoint the median tumor SUV_10_ tended to be lower for the treatment group (treatment 6.8 [5.5–7.9]; control: 11.0 [6.3–15.8]; 0 = 0.083). Furthermore, at the endpoint the median tumor SUV_10_ was significantly lower for the treatment group (3.9 [3.0-5.9]) compared to the control group (7.7 [5.4–10.6], *p* = 0.013), indicating treatment response. The longitudinal SUV_10_ fold change of individual tumors compared to baseline is shown in Fig. 4B. *P*-values from the paired analyses are given in Table 1.

In Model B, the median [^18^F]FDG tumor uptake was similar between the control- and treatment group both at baseline (control: 3.7 [3.5-5.0]; treatment: 3.8 [3.2–4.9], *p* = 0.684) and at the early timepoint (control: 5.8 [4.5–7.8]; treatment: 4.7 [2.7–6.5], *p* = 0.662) (Fig. 4C). At the endpoint (Day 10), the median tumor SUV_10_ was significantly decreased for the treatment group (3.6 [2.8–4.9]) compared to the control group (5.6 [4.4–6.7], *p* = 0.041), indicating treatment response. The SUV_10_ longitudinal fold change of individual tumors compared to baseline SUV_10_ is illustrated in Fig. 4D. Quantification of SUV_max_ displayed similar results to SUV_10_ in both models, with lower SUV_max_ in the treatment groups compared to the controls at the early timepoint, although not statistically significant (Supplementary Fig. 1).

### Early treatment response is detected by decreased metabolic rate of glucose (MR_FDG_) measured by dynamic [^18^F]FDG-PET in model A

The median [IQR] metabolic rate of [^18^F]FDG (MR_FDG_) quantified from the dynamic PET images was similar between the two groups in Model A at baseline (control: 1.55 [1.21–1.90] µmol*ml/g*min; treatment: 1.28 [4.0-9.7], *p* = 0.573), Fig. 4E. At the early timepoint (Day 3) and after 1 treatment cycle, the median MR_FDG_ was significantly lower in the treatment group (1.33 [0.52–1.66] µmol*ml/g*min) compared to the controls (2.21 [1.63–4.27], µmol*ml/g*min, *p* = 0.031). An identical pattern was observed at endpoint with lower median MR_FDG_ in the treatment group (1.12 [0.65–1.54] µmol*ml/g*min) compared to the control group (2.27 [1.21–6.57] µmol*ml/g*min, *p* = 0.042). MR_FDG_ longitudinal fold change of individual tumors compared to corresponding baseline MR_FDG_ is illustrated in Fig. 4F and *p*-values from pairwise analysis within the groups are shown in Table 1.

In Model B, median [IQR] tumor MR_FDG_ was similar between the two groups at baseline (control: 0.90 [0.79–1.14] µmol*ml/g*min; treatment: 1.08 [0.80–1.28] µmol*ml/g*min, *p* = 0.513) and at the early timepoint (control: 1.06 [0.73–1.79] µmol*ml/g*min; treatment: 1.39 [0.58–1.62] µmol*ml/g*min, *p* = 0.879). At endpoint, the median MR_FDG_ tended to be lower in the treatment group compared to the control, (control: 1.12 [0.60–1.51] µmol*ml/g*min; treatment: 0.84 [0.49–1.08] µmol*ml/g*min, *p* = 0.217). The MR_FDG_ longitudinal fold change of Model B individual tumors compared to baseline MR_FDG_ is illustrated in Fig. 4H.

Table 2 displays the correlation between the semi-quantitative static (SUV_10_) parameter and quantitative dynamic (MR_FDG_) parameter for both models, combining treatment- and control groups across the three timepoints.

**Table 2:** Correlation between metabolic parameters SUV_10_ and MR_FDG_ across all timepoints

	Model A (EEC G3)		Model B (EEC G3)	
	Correlation, *r* (95% CI)	*P*-value	Correlation, *r* (95% CI)	*P*-value
Baseline	0.689 (0.250–0.893)	*0.006*	0.866 (0.620–0.957)	*< 0.001*
Early	0.754 (0.319–0.927)	*0.005*	0.768 (0.401–0.923)	*0.001*
Endpoint	0.869 (0.599–0.962)	*< 0.001*	0.903 (0.683–0.973)	*< 0.001*

Correlation (Pearson *r*/Spearman *r* depending on dataset normality) between SUV_10_ and MR_FDG_ across the different timepoints, combining the control- and treatment groups in the two models

Statistically significant *p*-values (*p* < 0.05) in *italics*

**Abbreviations**: CI confidence interval, EEC G3 endometrioid endometrial cancer grade 3. MR_FDG_ metabolic rate of [^18^F]FDG, derived from dynamic imaging, SUV_10_ standardised uptake value of 10 hottest tumor voxels

### Endpoint treatment response is confirmed in tissue sections

The H&E tumor tissue sections from the endpoint confirmed the treatment effect at the cellular level in Model A (Supplementary Fig. 2). The mean [standard deviation, [SD]) cell density was significantly lower in the treatment group (9.6 [0.8] x10^3^ cells/mm^2^) compared to the control group (11.8 [1.1] x10^3^ cells/mm^2^, *p* = 0.001). In Model B, the mean cell density tended to be lower for the treatment group (8.7 [1.4] x10^3^ cells/mm^2^) than in the control group (10.0 [1.2] x10^3^ cells/mm^2^, *p* = 0.09). Difference in treatment response between the two models was also observed in in vitro experiments (Supplementary Fig. 3).

## Discussion

In this study we describe non-invasive, longitudinal [^18^F]FDG PET-MRI for assessment of early treatment response in two preclinical subcutaneous organoid models of human endometrial cancer treated with combined carboplatin and paclitaxel. Metabolic rate of glucose, MR_FDG,_ derived from dynamic PET was able to detect early treatment response (at Day 3) after treatment start, and only one cycle of carboplatin-paclitaxel in Model A, but not in Model B. No significant difference between the groups was detected by either static [^18^F]FDG-PET or DWI at the early timepoint in both models. The early response was evaluated against endpoint reduction in anatomical tumor volume (vMRI), which displayed significantly decreased vMRI in the treatment groups in both models. Overall, Model A had a more effective response to chemotherapy compared to Model B detected by all imaging parameters at the endpoint. Cell density in tumor tissue sections at endpoint in treated tumors versus controls were also significantly lower in Model A but not in Model B. The longitudinal individual tumor plots for vMRI and ADC_mean_ (Fig. 3) clearly showed a more heterogenous treatment response within Model B compared to Model (A) The individual treatment responses were also more heterogenous in Model B for the static and dynamic PET analyses (Fig. 4). Based on these results, Model A seems to be a better chemotherapy-responding model, whereas Model B may be classified as a mixed-response/resistant model, similar to what we have previously shown in vitro (Supplementary Fig. 3) [20]. This could also explain why MR_FDG_ was significantly decreased in the treatment group in Model A and not in Model (B) Endometrial cancer is highly heterogeneous [27], and patients are known to respond differently to chemotherapy with 40–60% reported chemoresistance [28, 29]. These preclinical organoid models thus seem to be highly relevant for capturing differences in chemotherapeutic responses that may be translated to a clinical setting. The mechanisms underlying chemotherapy resistance in endometrial cancer are poorly understood. Studies in other cancers have suggested apoptotic signaling, DNA-repair pathways, detoxification and autophagy as likely drivers [30, 31].

Similar to our results, dynamic [^18^F]FDG-PET tumor parameters in orthotopic breast cancer xenograft models after treatment with doxorubicin, carboplatin or paclitaxel was superior compared to static PET analyses (SUV) to assess treatment response [14]. Furthermore, a preclinical study investigating the treatment effect of radiation therapy on head-and-neck cancer xenografts, reported that the dynamic parameter tumor influx rate (K_i_) yielded better prediction of response than SUV markers [32]. Taken together, these results suggest that dynamic tumor PET markers may be more sensitive for detection of treatment response than static tumor PET markers. With recent improvements in hardware and software of clinical PET/CT scanners, including whole-body/long-axial field of view scanners, there is increasing interest in dynamic PET imaging [15–17]. Studies are now focusing on improving the clinical feasibility by using population-based input function and thereby decreasing the dynamic scan time from 70 min, to scan times of only 20 min (with dynamic imaging at 50 to 70 min post injection) [33]. Dynamic [^18^F]FDG-PET allows separation of bound [^18^F]FDG signal (K_i_/MR_FDG_ images) from the free reversible [^18^F]FDG signal (distribution volume, DV images), whereas standard static [^18^F]FDG-PET displays the combined bound and unbound signal (SUV images) [15]. This method may therefore improve detection of, and separation of metastatic disease from inflammatory/benign signal, in addition to increased quantification accuracy [15].

In a study using a mouse model of subcutaneous human ovarian cancer, Munk Jensen et al. showed decreased tumor SUV_mean_- and SUV_max_-ratios derived from static [^18^F]FDG-PET imaging 4 days after starting combined paclitaxel and carboplatin treatment [13]. In a similar preclinical ovarian cancer study, static [^18^F]FDG-PET (SUV_max_-ratio) on day 3 correlated with tumor sizes at day 10 following anti-tumor treatment with the histone deacetylase inhibitor belinostat [34]. In a subcutaneous triple negative breast cancer mouse model, metabolic tumor volume from static [^18^F]FDG-PET was significantly decreased in responding versus non-responding mice 3 days after treatment with cisplatin [10]. Albeit applying different PET parameters, all these studies detected a metabolic ([^18^F]FDG) response prior to a decrease in tumor size (measured by CT or calipers), similar to our findings (MR_FDG_) in Model A. Reduction in metabolic activity may precede reduction in tumor size [23, 35, 36], and encourages the use of [^18^F]FDG-PET imaging for evaluation of treatment response following chemotherapy. Most targeted effective cancer treatments do not necessarily result in immediate tumor shrinkage, thus establishing alternative early markers of response, e.g. change in tumor metabolism, may prove highly important, also in endometrial cancer.

In a subcutaneous model of triple negative breast cancer, Barnes et al. showed significantly decreased MRI-derived tumor volume day 3 after treatment with albumin-bound paclitaxel, and significantly increased median ADC-values already at Day 1 post-treatment [12]. In a similar breast cancer model, Nguyen-Thu et al. showed increased ADC_mean_ in cisplatin-responding mice Day 3 post-treatment [10]. In the present study we were not able to detect an early treatment response (Day 3) by DW-imaging. The individual tumor plots from DW-MRI indicate that even though the endpoint effect was seemingly unanimous within the groups, there was considerable variation at the early timepoint. Additionally, it is possible that our study design with imaging at Day 3 post-treatment was a suboptimal timing for detecting early response in our models, and it is possible that altering the timepoint by 1–2 days would have yielded a different result.

The current study has some limitations. Although dynamic PET analyses may give more detailed information on uptake characteristics, these analyses can be technically challenging, and they depend on input from both tumor and blood pool. Compared to static PET with one long time frame, dynamic PET acquisitions are divided in multiple shorter time frames. This leads to a lower signal-to-noise ratio and may result in increased variability in analyses [37]. To reduce variation in static PET, we introduced the SUV_10_ parameter (average uptake of the 10 hottest tumor voxels) as an alternative to the standard SUV_max_ (the single hottest tumor voxel). SUV_10_ is therefore considered less susceptible to noise and partial volume effect.

## Conclusions

MR and PET sequential imaging is feasible for evaluation of very early treatment response in subcutaneous preclinical endometrial cancer models. The metabolic rate of glucose, MR_FDG_, derived from dynamic [^18^F]FDG-PET represents the most promising marker for detecting early (at Day 3) treatment response following chemotherapy.

## Electronic supplementary material

Below is the link to the electronic supplementary material.

Supplementary Material 1

## Data Availability

The datasets used and/or analysed during the current study are available from the corresponding author on reasonable request.

## Abbreviations

ADC: Apparent diffusion coefficient
Carbo-PTX: Carboplatin and Paclitaxel
DWI: Diffusion-weighted imaging
EEC G3: Endometrioid endometrial cancer grade 3
[^18^F]FDG: Fluorine-18 fluorodeoxyglucose
IQR: Interquartile range
MBq: Megabecquerel
MR_FDG_: Metabolic rate of ^18^F-FDG derived from dynamic PET
MRI: Magnetic Resonance Imaging
NA: Not applicable
ns: Not significant
O-PDX: Organoid-based patient-derived xenograft model
PET: Positron Emission Tomography
ROI: Region of interest
SD: Standard deviation
SUV: Standardised uptake value
SUV_10_: Average SUV of 10 hottest voxels
TE: Echo time
TR: Repetition time
vMRI: Tumor volume derived from MRI
VOI: Volume of interest
