# Supplementary material for: Evaluation of early chemotherapy response by combining static- and dynamic [18F]FDG-PET with diffusion-weighted MRI in subcutaneous patient-derived endometrial cancer mouse models
Source: EJNMMI Res. 2025 Apr 18;15:45. doi: 10.1186/s13550-025-01235-5 (PMC12008091; doi:10.1186/s13550-025-01235-5)

**
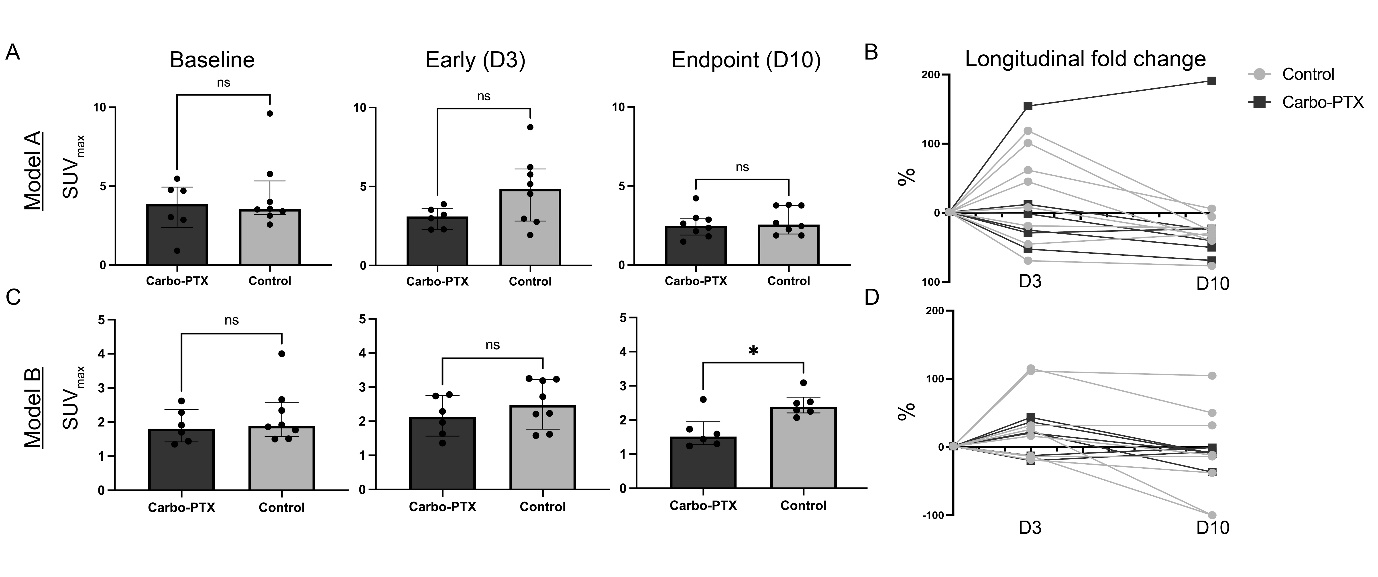
Supplementary Table 1:** Overview of number of tumors included for analyses per model, group and timepoint.

|  | Baseline (Day 0) | | Early (Day 3) | | Endpoint (Day 10) | |
| --- | --- | --- | --- | --- | --- | --- |
| EEC G3  Model | Total number of O-PDX tumors (Control tumors) | | | | | |
|  | A | B | A | B | A | B |
| T2w-MRI | 16 (8) | 14 (8) | 16 (8) | 14 (8) | 15 (7) | 14 (8) |
| DW-MRI | 16 (8) | 14 (8) | 16 (8) | 14 (8) | 13 (5) | 14 (8) |
| Static PET | 12 (8) | 14 (8) | 12 (8) | 14 (8) | 12 (6) | 12 (6) |
| Dynamic PET | 14 (8) | 14 (8) | 12 (6) | 14 (8) | 14 (6) | 12 (6) |
| Cell density | *NA* | | | | 14 (7) | 14 (8) |

**Abbreviations**: DW diffusion-weighted, EEC G3 endometrioid endometrial cancer grade 3, MRI magnetic resonance imaging, NA not applicable PET positron emission tomography, T2w T2-weighted

**Additional File 2 (JPG-file):**

**Supplementary Figure 1 – Quantification of SUV_max_ from static [^18^F]FDG-PET**

Quantification of SUV_max_ derived from the PET images for EEC G3 Model A (A-B) and EEC G3 Model B (C-D) for the different timepoints. Median and interquartile range are displayed for the bar graphs. Statistical significance (p-value <0.05) is indicated by *. The fold change (%) was calculated from the baseline values.

**Abbreviations**: Carbo-PTX Carboplatin-paclitaxel, D day, EEC G3 endometrioid endometrial cancer grade 3, [^18^F]FDG fluorodeoxyglucose, ns not significant, SUV_max_ standardised uptake value from the hottest tumor voxel

**
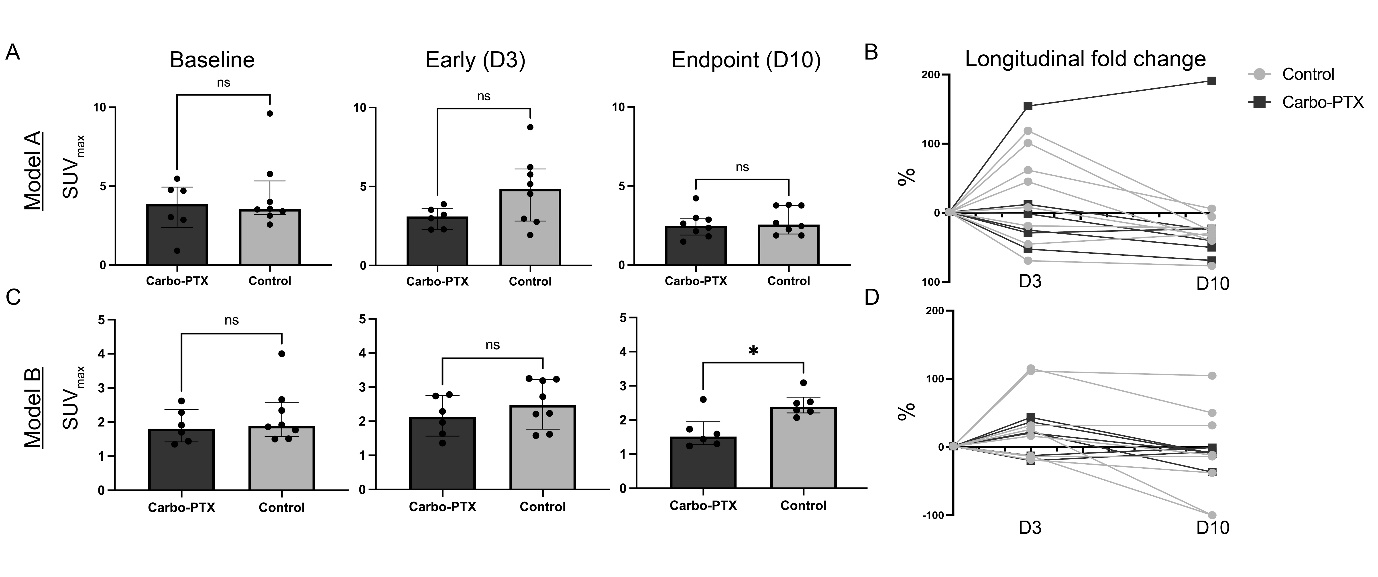
**

**Additional File 3 (JPG-file):**

**Supplementary Figure 2 - Cell density at endpoint**

Quantification of tumor cells from tissue sections collected immediately after the endpoint imaging session for EEC G3 Model A (A) and Model B (B). Cell density (cells/mm^2^) values are displayed as mean and standard deviation. Statistical significance (p-value <0.05) is indicated by *.

**Abbreviations** Carbo-PTX Carboplatin-paclitaxel, EEC G3 endometrioid endometrial cancer grade 3, ns not significant


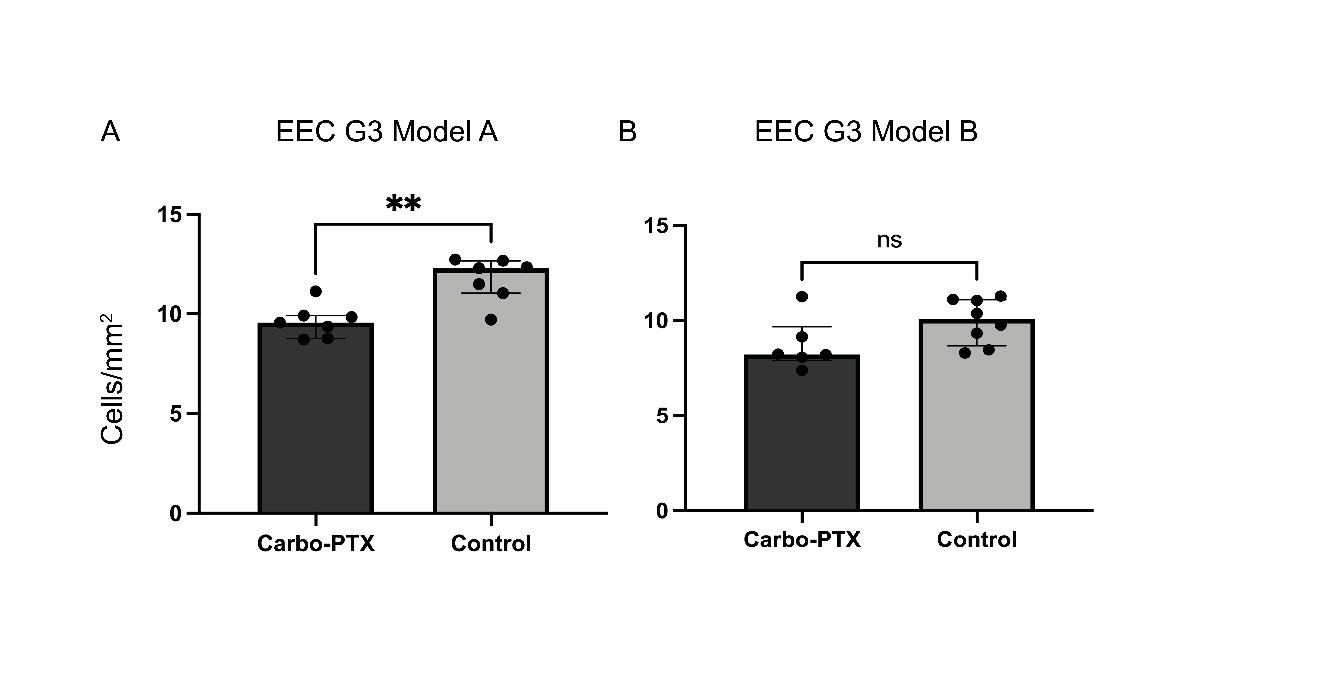


**Additional File 4 (JPG-file):**

**Supplementary Figure 3: In vitro treatment response**

In vitro treatment response in EEC G3 organoid Model A and Model B. Organoids were treated with 100uM/200uM Carboplatin (Carbo) combined with 200nM Paclitaxel (PTX) for 24 hours. Viability was measured with CellTiter-Glo 3D Assay. Standard deviation was calculated from three or more independent experiments (black dot). Corresponding OEC-07-G3 and OEC-11-G3 IDs and data was retrieved from Berg et al. (2021).(21).

**Abbreviations**: Carbo-PTX Carboplatin-paclitaxel, EEC G3 endometrioid endometrial cancer grade 3


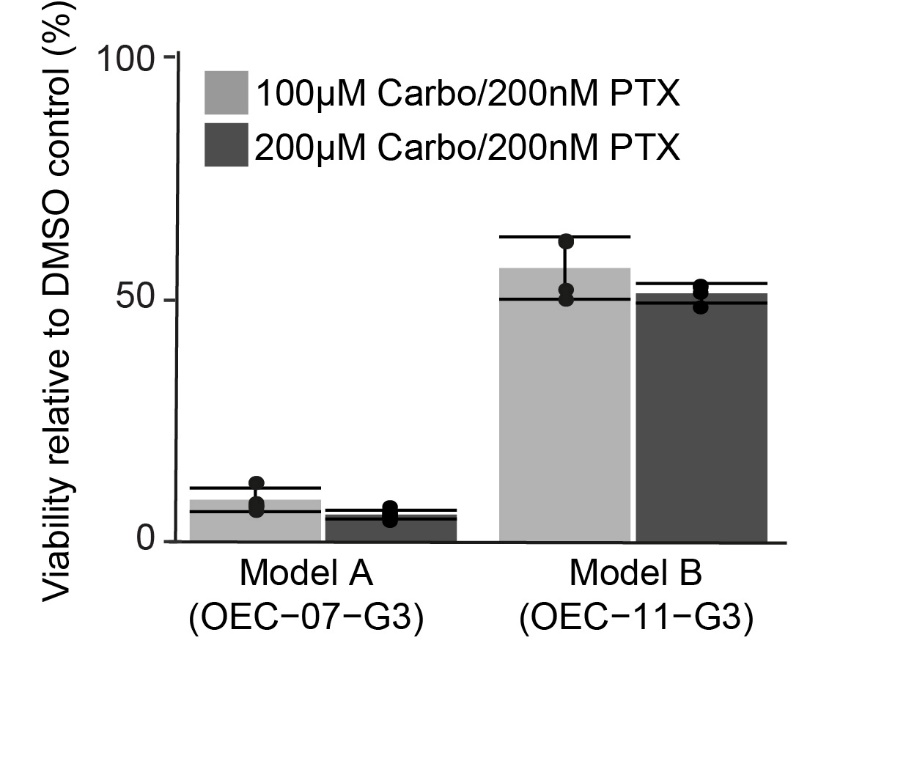

Supplement: Supplementary file 1 — Supplementary Material 1 [file 13550_2025_1235_MOESM1_ESM.docx]
